# Tanshinone IIA alleviates LPS-induced acute kidney injury by inhibiting RIP3/Nrf2-mediated oxidative stress

**DOI:** 10.1080/0886022X.2025.2593719

**Published:** 2025-12-04

**Authors:** Shu Zhang, Xin Dong, Gangyi Chen, Chao Wang

**Affiliations:** Department of Nephrology, The First Affiliated Hospital of Guangzhou University of Traditional Chinese Medicine, Guangzhou, China

**Keywords:** Tanshinone IIA, acute kidney injury, RIP3, oxidative stress, Nrf2

## Abstract

**Background:**

Oxidative stress is a critical pathological mechanism in septic acute kidney injury (AKI). Tanshinone IIA (Tan IIA), a bioactive compound derived from *Salvia miltiorrhiza*, exhibits antioxidative properties. This study aimed to explore the protective mechanisms of Tan IIA in septic AKI.

**Methods:**

A mouse model of lipopolysaccharide (LPS)-induced AKI was established, with Tan IIA administered prior to LPS injection. Renal function was assessed by serum creatinine and urea nitrogen levels, while histopathological damage and apoptosis were evaluated using PAS and TUNEL staining. Oxidative stress was assessed by SOD activity, ROS levels, and 8-OHdG staining. *In vitro*, LPS-stimulated HK-2 cells were treated with Tan IIA or RIP3 siRNA. The expression of RIP3, p-RIP3, Nrf2, HO-1, KEAP1, and downstream antioxidant genes (NQO1, GCLC) was analyzed by qRT-PCR, immunohistochemistry, and Western blot. Apoptosis was further evaluated by Annexin V-FITC/PI flow cytometry and detection of apoptosis-related proteins (Bax, cleaved caspase-3, Bcl-2). Co-immunoprecipitation was performed to examine RIP3–Nrf2 interaction.

**Results:**

*In vivo*, Tan IIA significantly improved renal function and alleviated tubular damage. It decreased ROS production and 8-OHdG positivity while restoring SOD activity. *In vitro*, Tan IIA or RIP3 silencing decreased ROS levels, enhanced SOD activity, promoted Nrf2 nuclear translocation, and upregulated antioxidant genes. Flow cytometry confirmed reduced apoptotic cell populations, and Western blot showed reversal of LPS-induced pro-apoptotic protein changes. The co-immunoprecipitation confirmed an interaction between RIP3 and Nrf2 under LPS stimulation.

**Conclusion:**

These findings suggest that Tan IIA protects against LPS-induced AKI by inhibiting RIP3 expression and restoring Nrf2-mediated antioxidant defenses, thereby attenuating oxidative stress and apoptosis.

## Introduction

Sepsis-associated acute kidney injury (AKI) is a severe clinical condition characterized by a rapid decline in renal function due to infection-triggered inflammatory and oxidative responses [1,2]. Despite advances in treatment strategies, the morbidity and mortality of septic AKI remain alarmingly high, reflecting the complexity of its pathophysiological mechanisms and the lack of effective therapeutic interventions [3,4]. Oxidative stress, driven by the excessive production of reactive oxygen species (ROS) and impaired antioxidant defenses, is a critical driver of cellular injury in septic AKI [4,5]. These insights emphasize the need for novel strategies to mitigate oxidative stress and protect renal tubular epithelial cells during sepsis.

Tanshinone IIA (Tan IIA), a bioactive compound derived from the traditional Chinese medicinal herb Salvia miltiorrhiza, is renowned for its potent antioxidant, anti-inflammatory, and free radical-scavenging properties [6–8]. Emerging studies suggest that Tan IIA can improve renal outcomes by enhancing cell­ular antioxidant capacity and mitigating oxidative damage [9]. Notably, Tan IIA has been shown to activate the nuclear factor erythroid 2-related factor 2 (Nrf2) signaling pathway, a critical regulator of antioxidant responses that governs the expression of genes such as heme oxygenase-1 (HO-1) and NAD(P)H quinone dehydrogenase 1 (NQO1) [10,11]. However, the precise mechanisms by which Tan IIA modulates Nrf2 activation to confer renal protection remain insufficiently understood.

Recent evidence highlights the role of receptor-interacting protein kinase 3 (RIP3) in mediating oxidative stress, inflammation, and cell death in septic AKI [12]. RIP3, a key regulator of necroptosis, has been implicated in exacerbating oxidative damage and apoptosis in renal tubular cells [13,14]. Experimental models have demonstrated that inhibiting RIP3 can alleviate septic kidney injury by reducing mitochondrial dysfunction and oxidative stress [15]. However, the interplay between RIP3 and Nrf2 signaling pathways in the context of AKI remains unclear, and whether Tan IIA exerts its protective effects through modulating RIP3 activity has yet to be elucidated.

This study investigates the potential of Tan IIA to alleviate septic AKI by targeting the RIP3/Nrf2 signaling axis. We hypothesized that Tan IIA inhibits RIP3 activation, thereby enhancing Nrf2 nuclear translocation and promoting antioxidant defense mechanisms. By employing both *in vivo* and *in vitro* models, this research aims to provide novel insights into the molecular mechanisms underlying Tan IIA’s protective effects and its potential therapeutic applications for septic AKI. The findings of this study are anticipated to offer significant contributions to the understanding of septic AKI pathophysiology and the development of integrative treatment approaches combining traditional Chinese medicine with modern therapeutic strategies.

## Materials and methods

### Animal experiments

Thirty 12-week-old male C57BL/6 mice were purchased from Guangzhou Yongnuo Biotechnology Co., Ltd (Guangzhou, Guangdong) and maintained in a specific pathogen-free (SPF) environment at 22 °C–26 °C. The animal experiments were approved by the Ethics Committee of Guangzhou University of Chinese Medicine. The mice were randomly divided into three groups (*n* = 10 per group): control, LPS, and LPS+Tan IIA groups. The control group received an intraperitoneal injection of saline, while the LPS group was injected with 10 mg/kg of lipopolysaccharide (LPS, *Escherichia coli O55:B5*, HY-D1056, MedChemExpress, USA) [16]. In the LPS+Tan IIA group, mice were pretreated with 10 mg/kg [17] of Tan IIA (purity > 98%; Aladdin, Shanghai, China) *via* intraperitoneal injection 3 h before receiving 10 mg/kg of LPS. To monitor physiological parameters, mice were placed in metabolic cages 72 h prior to intervention, where body weight, urine output, water intake, and food consumption were recorded. Twenty-four hours after LPS or saline administration, mice were anesthetized pentobarbital sodium (50 mg/kg, intraperitoneally). Adequate anesthesia was confirmed by the absence of pedal withdrawal reflex. Blood samples were then collected from the inferior vena cava, and bilateral kidneys were harvested. Mice were subsequently euthanized by exsanguination under deep anesthesia, in accordance with protocols approved by the Ethics Committee of Guangzhou University of Chinese Medicine (Approval No. IACUC-AEWC-F2109012) and following the AVMA Guidelines for the Euthanasia of Animals (2020 edition).

### Cell culture and transfection

Human renal tubular epithelial cells (HK-2) cells were purchased from the American Tissue Culture Collection (Rockville MD, USA). The cells were cultured in a DMEM/F12 medium (Thermo Fisher Scientific, Waltham, MA, USA) supplemented with 10% fetal bovine serum (Thermo Fisher Scientific) in a cell culture incubator (37 °C, 5% CO_2_). The medium was changed every 2–3 days, and cells were passaged when their confluency reached approximately 90%. The cells were divided into five groups, namely the control, LPS (LPS 10 µg/mL [18] for 24 h; MilliporeSigma), LPS+Tan IIA (LPS 10 µg/mL + Tan IIA 5 µM 19], LPS+siNC (LPS 10 µg/mL + 50 nM siNC), and LPS+siRIP3 groups (LPS 10 µg/mL+ 50 nM siRIP3). Tan IIA reagents (purity > 98%) was purchased from Aladdin (Shanghai, China).

For RIP3 knockdown, cells were transfected with RIP3 small interfering RNA (siRNA) (RiboBio, Guangzhou, China). The cell culture medium was discarded, and the cells were washed three times with PBS. Then, an appropriate amount of serum-free DMEM/F12 medium was added. The siRNA-Lipo2000 (Thermo Fisher Scientific, Scotts Valley, CA, USA) mixture was thereupon added to the culture medium, and the dish was inserted in a cell culture incubator (37 °C, 5% CO_2_). After 6 to 8 h, transfection efficiency was assessed. Following successful transfection, the medium containing 10% FBS was replaced.

### Measurement of blood creatinine, blood urea nitrogen, SOD, and MDA levels

To evaluate the renal function of the mice, the QuantiChromTM Creatinine Assay kit (BioAssay Systems) and the QuantiChromTM blood urea nitrogen (BUN) Assay kit (BioAssay Systems) were used to measure blood creatinine and blood urea nitrogen levels. SOD activity in kidney tissues and cell supernatant was determined using Superoxide Dismutase (SOD) Activity Assay Kit (Najing Jiancheng Bioengineering Institute, Najing, China, Cat. No. A001-3) through the WST-1 method according to the manufacturer’s instructions. Intracellular reactive oxygen species (ROS) was detected using Reactive Oxygen Species Assay Kit (Beyotime Biotechnology, Shanghai, China, Cat. No. S0033S). Hydroxyl Free Radical assay kit was provided from Najing Jiancheng Bioengineering Institute (Najing, China, Cat. No. A018-1).

### Periodic acid-Schiff (PAS) staining of kidney tissues

Kidney tissues were fixed in 4% paraformaldehyde and dehydrated through a graded ethanol series (70%, 80%, 90%, 95%, and 100%) followed by xylene. The tissues were embedded in paraffin, sectioned into 4-μm slices, and deparaffinized with xylene. After rehydration, the sections were treated with 1% periodic acid solution (Cat. No. ab150680, Abcam) for 10 min and rinsed with double-distilled water. Subsequently, the sections were exposed to Schiff’s reagent (Cat. No. ab150680, Abcam) for 20 min at room temperature in the dark, followed by thorough washing with double-distilled water. Hematoxylin was used for nuclear counterstaining, and the sections were dehydrated, cleared with xylene, and sealed with neutral balsam (Cat. No. G8590, Solarbio Science & Technology Co., Ltd., Beijing, China). Slides were visualized under a light microscope at 200 × magnification.

For histopathological analysis, 10 random fields per section were examined at 200 × magnification. Renal tubular injury was scored based on the extent of pathological changes, including tubular lumen narrowing, brush border detachment, vacuolar degeneration, epithelial swelling, and basement membrane exposure. Injury severity was graded as follows: 0 = no injury; 1 = injury in ≤ 25% of the area; 2 = injury in 25%–50% of the area; 3 = injury in 50%–75% of the area; and 4 = injury in > 75% of the area [20].

### 8-OHdG staining of kidney tissues

Kidney tissue sections (4 μm) were deparaffinized in xylene and rehydrated through a graded ethanol series. After washing with PBS, the sections were incubated in 1% H2O2 for 10 min at room temperature to block endogenous peroxidase activity. To detect oxidative DNA damage, sections were incubated with a primary antibody against 8-hydroxy-2′-deoxyguanosine (8-hydroxydeoxyguanosine Assay Kit, China, Cat. No.H165-1-1, Najing Jiancheng Bioengineering Institute Najing) at 4 °C overnight. Following washing with PBS, sections were incubated with a biotinylated secondary antibody for 1 h at room temperature. The reaction was visualized using an avidin-biotin complex (ABC) kit and diaminobenzidine (DAB) as a chromogen. After counterstaining with hematoxylin, sections were dehydrated, cleared, and mounted with neutral balsam. The stained sections were observed under a light microscope (magnification ×200). The number of 8-OHdG-positive cells was quantified in 10 randomly selected fields per section and expressed as the percentage of positive cells relative to the total number of cells.

### TUNEL assay

To evaluate apoptosis in mouse renal tissues, a terminal deoxynucleotidyl transferase-mediated dUTP nick end labeling (TUNEL) assay was performed using a commercially available TUNEL kit following the manufacturer’s protocol. Briefly, paraffin-embedded kidney sections (4 μm) were prepared and deparaffinized with xylene, followed by rehydration in graded ethanol. Tissue permeabilization was performed using proteinase K (20 μg/mL) at 37 °C for 20 min. After quenching endogenous peroxidase with 3% hydrogen peroxide, sections were incubated with the TUNEL reaction mixture containing terminal deoxynucleotidyl transferase (TdT) and fluorescein-labeled dUTP in a humidified chamber at 37 °C for 1 h. Negative controls were prepared without TdT enzyme. After washing with PBS, nuclei were counterstained with DAPI, and TUNEL-positive cells (green fluorescence) were visualized and quantified using a fluorescence microscope (200 × magnification). Ten random fields per section were analyzed to determine apoptotic cell numbers.

### Western blot

Kidney tissues or cultured cells were lysed using a radioimmunoprecipitation assay (RIPA) buffer (Beyotime Biotechnology, Shanghai, China) supplemented with protease and phosphatase inhibitors (Roche, Basel, Switzerland). The lysates were centrifuged at 12,000 × g for 15 min at 4 °C to collect the supernatant containing total protein. Nuclear and cytoplasmic proteins were extracted separately using a nuclear protein extraction kit (CelLytic^™^ NuCLEAR Extraction Kit, MilliporeSigma, Burlington, MA, USA) according to the manufacturer’s instructions. Protein concentration was quantified using the bicinchoninic acid (BCA) protein assay kit (Thermo Fisher Scientific, Waltham, MA, USA). Equal amounts of protein (20 μg) were mixed with loading buffer, denatured by boiling at 95 °C for 5 min, and separated on SDS-PAGE gels (12% polyacrylamide, depending on target protein size). Proteins were transferred onto polyvinylidene difluoride (PVDF) membranes (MilliporeSigma) using the wet transfer method. The membranes were blocked with 5% nonfat milk or 5% bovine serum albumin (BSA) in Tris-buffered saline containing 0.1% Tween-20 (TBST) for 1 h at room temperature to prevent nonspecific binding.

Blocked membranes were incubated overnight at 4 °C with primary antibodies. After washing with TBST, membranes were incubated with HRP-conjugated secondary antibodies (1:5,000) for 1 h at room temperature. Protein bands were visualized using an enhanced chemiluminescence (ECL) detection system (Thermo Fisher Scientific) and imaged with a gel documentation system. All experiments were repeated at least three times independently.

The following primary antibodies were used: anti-GAPDH antibody (1:10000 dilution; Cat. no. 60004-1-lg; Proteintech Group, Inc., Wuhan, China), anti-Histone antibody (1:10000 dilution; Cat. no. 17168-1-AP; Proteintech Group, Inc.), anti-KEAP1 antibody, (1:5000 dilution; Cat. no. 10503-2-AP; Proteintech Group, Inc.), anti-HO-1 antibody, (1:1000 dilution; Cat. no. 70081; Cell Signaling Technology, Inc.), anti-RIP3 antibody (1:1000 dilution; Cat. no. 17563-1-AP; Proteintech Group, Inc.), anti-Nrf2 antibody (1:1000 dilution; Cat. no. bs-1074R; Bioss, Beijing, China), anti-phosphorylated RIP3 antibody (p-RIP3) (1:1000 ­dilution; Cat. no. ab195117, Abcam), anti-phosphorylated RIP3 antibody (p-RIP3) (1:1000 dilution; Cat. no. ab209384, Abcam).

### Flow cytometry analysis of apoptosis

Apoptosis in HK-2 cells was assessed using an Annexin V-FITC/propidium iodide (PI) apoptosis detection kit (Beyotime Biotechnology, Shanghai, China) according to the manufacturer’s instructions. Briefly, after the indicated treatments, cells were harvested, washed twice with cold phosphate-buffered saline (PBS), and resuspended in 1× binding buffer. Cells (1 × 10^5^) were stained with 5 μL of Annexin V-FITC and 5 μL of PI for 15 min at room temperature in the dark. Samples were analyzed using a flow cytometer (BD FACSCanto II, BD Biosciences, USA), and data were processed with FlowJo software (Tree Star Inc., USA). The percentage of early and late apoptotic cells was calculated and used to assess apoptosis levels.

### Co-immunoprecipitation

Co-immunoprecipitation (Co-IP) experiments were conducted using the Dynabeads Protein G Immunoprecipitation Kit (Thermo Fisher Scientific) in accordance with the manufacturer’s instructions. HK-2 cells were washed twice with ice-cold PBS and lysed using Pierce IP Lysis Buffer (87787; Thermo Fisher Scientific) supplemented with protease inhibitor (Beyotime Biotechnology). To reduce nonspecific binding, the lysates were pre-cleared with Dynabeads Protein G. Subsequently, the lysates were incubated with an appropriate amount of anti-Nrf2 antibody (catalog number ab137550; Abcam), anti-RIP3 antibody (catalog number 17563-1-AP; Proteintech), or control IgG at 4 °C overnight. The antibody-antigen complexes bound to Dynabeads were washed with wash buffer and eluted using RIPA buffer for subsequent Western blot analysis.

### Statistical analysis

Statistical analyses were performed using SPSS 20.0 software. All quantitative data were subjected to the normality test and chi-square test. Variables that conformed to a normal distribution were expressed as mean ± standard deviation (SD). One-way analysis of variance (ANOVA) was used for multiple group comparisons, and Bonferroni’s multiple comparison method was used for two-by-two comparisons between groups. *p* < 0.05 was considered statistically significant.

## Results

### Tanshinone IIA preserves renal function and mitigates histopathological damage in LPS-induced AKI

**Figure 1.**
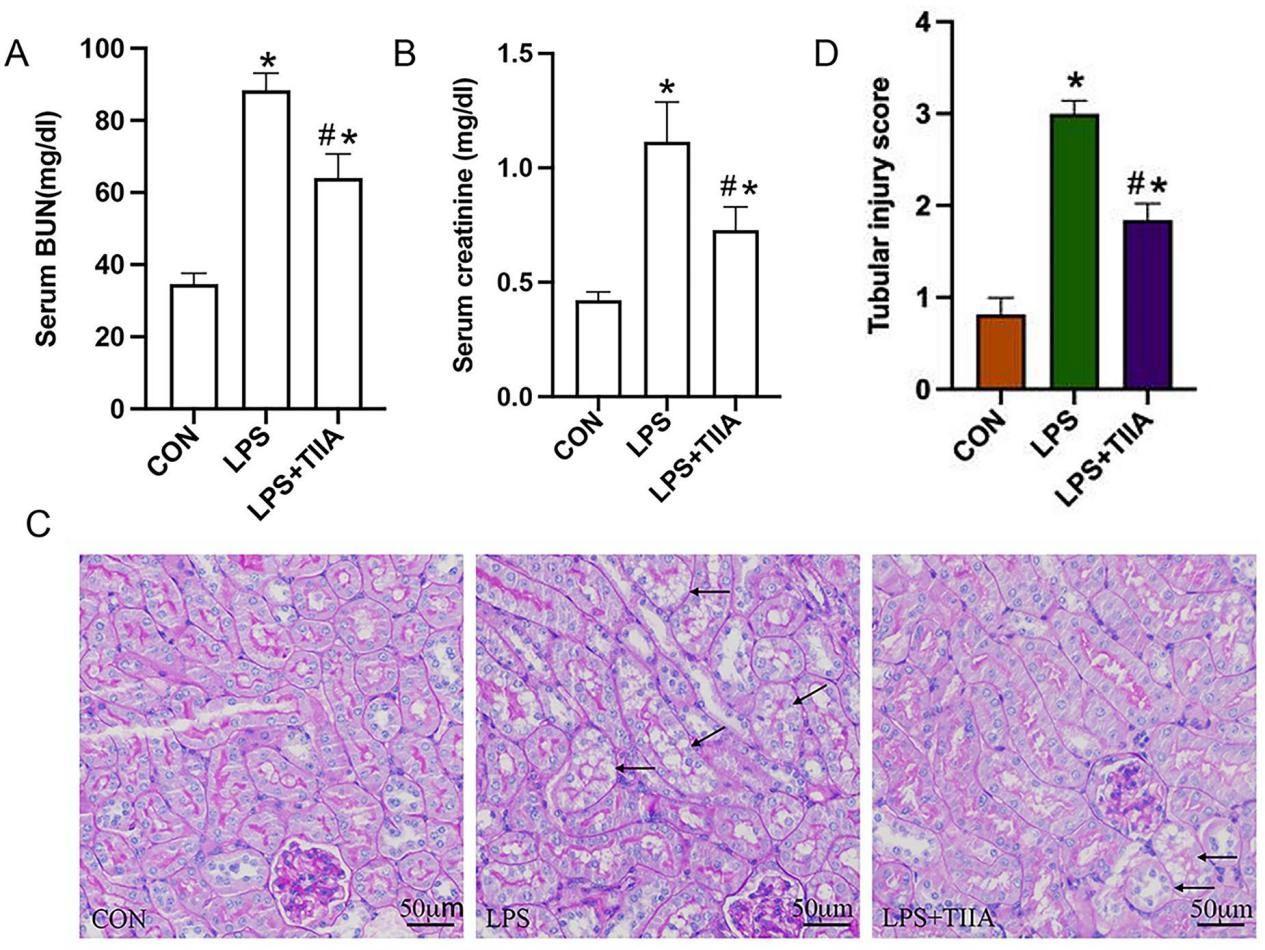
Effect of Tanshinone IIA on renal function and histopathology in LPS-induced AKI. (A and B): Blood urea nitrogen (A) and creatinine (B) levels across treatment groups (Control, LPS, LPS + Tan IIA). (C) Representative PAS-stained renal tissue sections. (D) Renal tubular injury scores in each group. *P < 0.05 *vs*. Control group, #*P* < 0.05 *vs*. LPS group. LPS, lipopolysaccharide; Tan IIA, Tanshinone IIA; PAS, Periodic Acid-Schiff stain.

In the LPS-induced AKI model, treatment with Tan IIA significantly improved renal function as indicated by reduced blood urea nitrogen and creatinine levels (Figure 1A,B). Histopathological analysis of renal tissue using PAS staining revealed pronounced tubular damage, including vacuolar degeneration and epithelial swelling, in the LPS group. Notably, Tan IIA administration attenuated these morphological abnormalities, suggesting a protective effect against LPS-induced kidney injury (Figures 1C,D).

### Tanshinone IIA reduces oxidative stress in renal tissue

**Figure 2.**
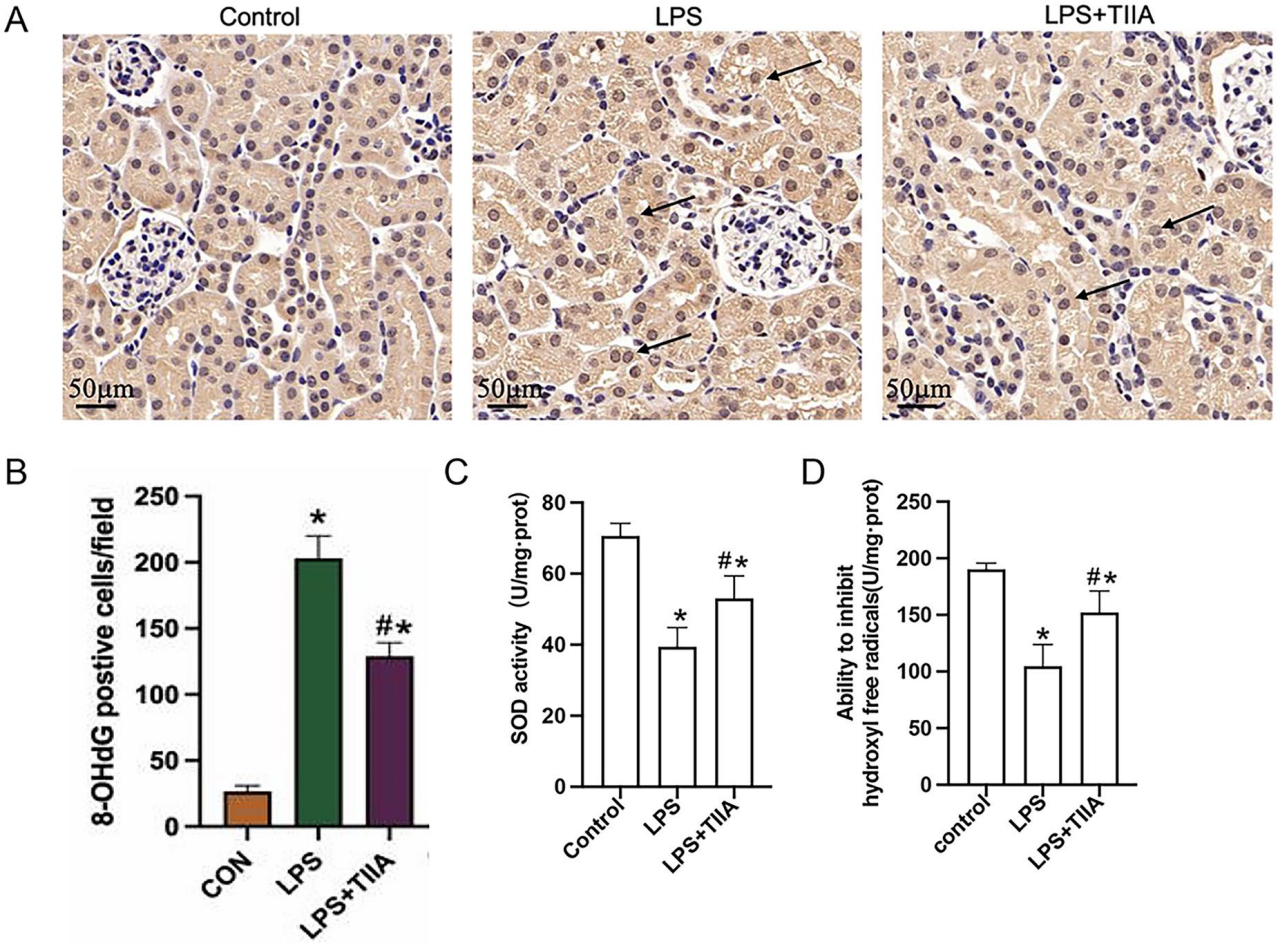
Antioxidant effects of Tan IIA in renal tissue. (A) Representative 8-OHdG staining images. (B) Quantitative analysis of 8-OHdG-positive cells in kidney sections. (C and D): SOD activity (C) and hydroxyl radical inhibition levels (D) in kidney tissues of each group. *P < 0.05 *vs*. Control group, #*P* < 0.05 *vs*. LPS group. LPS, lipopolysaccharide; Tan IIA, Tanshinone IIA; 8-OHdG, 8-hydroxy-2’-deoxyguanosine; SOD, Superoxide dismutase.

To assess the antioxidative effects of Tan IIA, we evaluated oxidative stress indicators in renal tissue. Immunohistochemical staining of 8-OHdG, a marker of oxidative DNA damage, showed increased positive staining in the LPS group compared to the control, which was significantly reduced in the Tan IIA-treated group (Figure 2A,B). LPS-induced oxidative stress significantly decreased SOD activity and the ability to inhibit hydroxyl radicals in renal tissues. These oxidative stress indicators were restored by Tan IIA treatment, indicating enhanced antioxidant capacity (Figure 2C,D). These findings suggest that Tan IIA effectively mitigates oxidative stress in LPS-induced AKI.

### Tan IIA reduces apoptosis and regulates the RIP3/Nrf2 pathway in LPS-induced AKI

**Figure 3.**
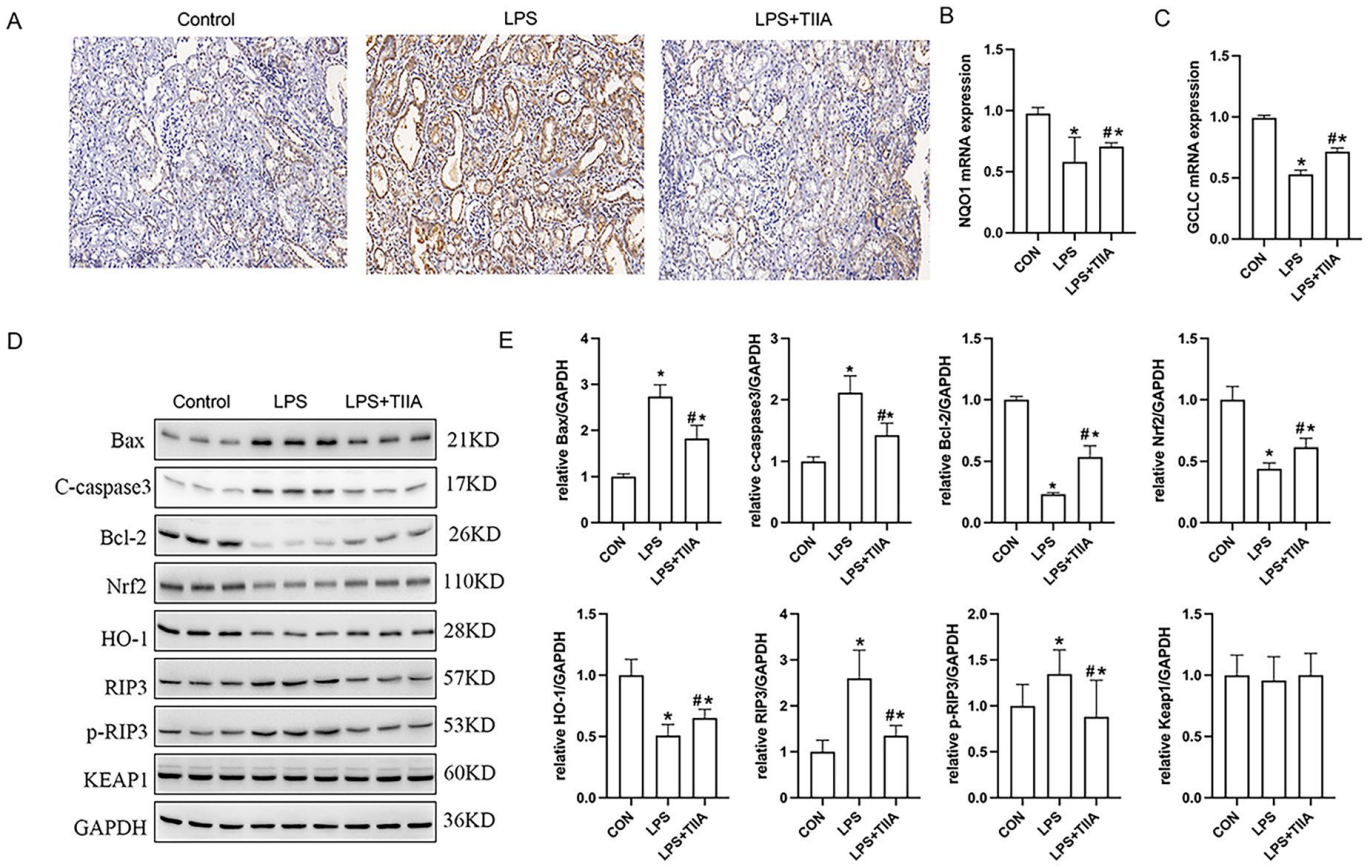
Effects of Tan IIA on apoptosis and RIP3/Nrf2 pathway in LPS-induced AKI. (A) TUNEL staining in kidney tissues from the control, LPS, and LPS+Tan IIA groups. (B and C) The mRNA expressions of NQO1 (B) and GCLC (C) in renal tissues were detected by RT-qPCR. (D and E) Western blot analysis of apoptosis-related proteins (Bax, cleaved caspase-3, Bcl-2), RIP3/p-RIP3, and Nrf2 pathway markers (Nrf2, HO-1, and KEAP1). *P < 0.05 *vs*. Control; #*P* < 0.05 *vs*. LPS group. TUNEL, terminal deoxynucleotidyl transferase-mediated dUTP nick end labeling; LPS group. LPS, lipopolysaccharide; Tan IIA, Tanshinone IIA; RIP3, receptor-interacting protein kinase 3; Nrf2, Nuclear factor erythroid 2-related factor 2; HO-1, Heme oxygenase-1; KEAP1, Kelch-like ECH-associated protein 1, NQO1, NAD(P)H quinone dehydrogenase 1; GCLC, Glutamate-cysteine ligase catalytic subunit.

Further we explored the mechanism of the protective effect of Tan IIA against renal injury. TUNEL staining showed a significant increase in apoptotic cells in the LPS group compared to the control, while Tan IIA treatment significantly reduced apoptosis (Figure 3A). Additionally, qRT-PCR analysis demonstrated that LPS significantly downregulated the mRNA expression of NQO1 and GCLC, two classical Nrf2 target genes (Figure 3B,C). Treatment with Tan IIA effectively reversed these reductions.

Western blot analysis further confirmed these effects (Figure 3D,E). LPS exposure led to a substantial increase in the expression of pro-apoptotic proteins Bax and cleaved caspase-3, as well as RIP3, p-RIP3, and KEAP1, along with a decrease in Bcl-2, HO-1, and Nrf2 expression. Tan IIA treatment reversed these alterations by reducing the levels of Bax, cleaved caspase-3, RIP3, p-RIP3, and KEAP1, while restoring Bcl-2, HO-1, and Nrf2 levels.

These data collectively suggest that Tan IIA attenuates oxidative stress and apoptosis in LPS-induced AKI through inhibition of RIP3 and activation of the Nrf2/HO-1 antioxidant pathway.

### Tan IIA modulates RIP3/Nrf2 pathway to protect against oxidative stress and apoptosis in LPS-induced HK-2 cells

**Figure 4.**
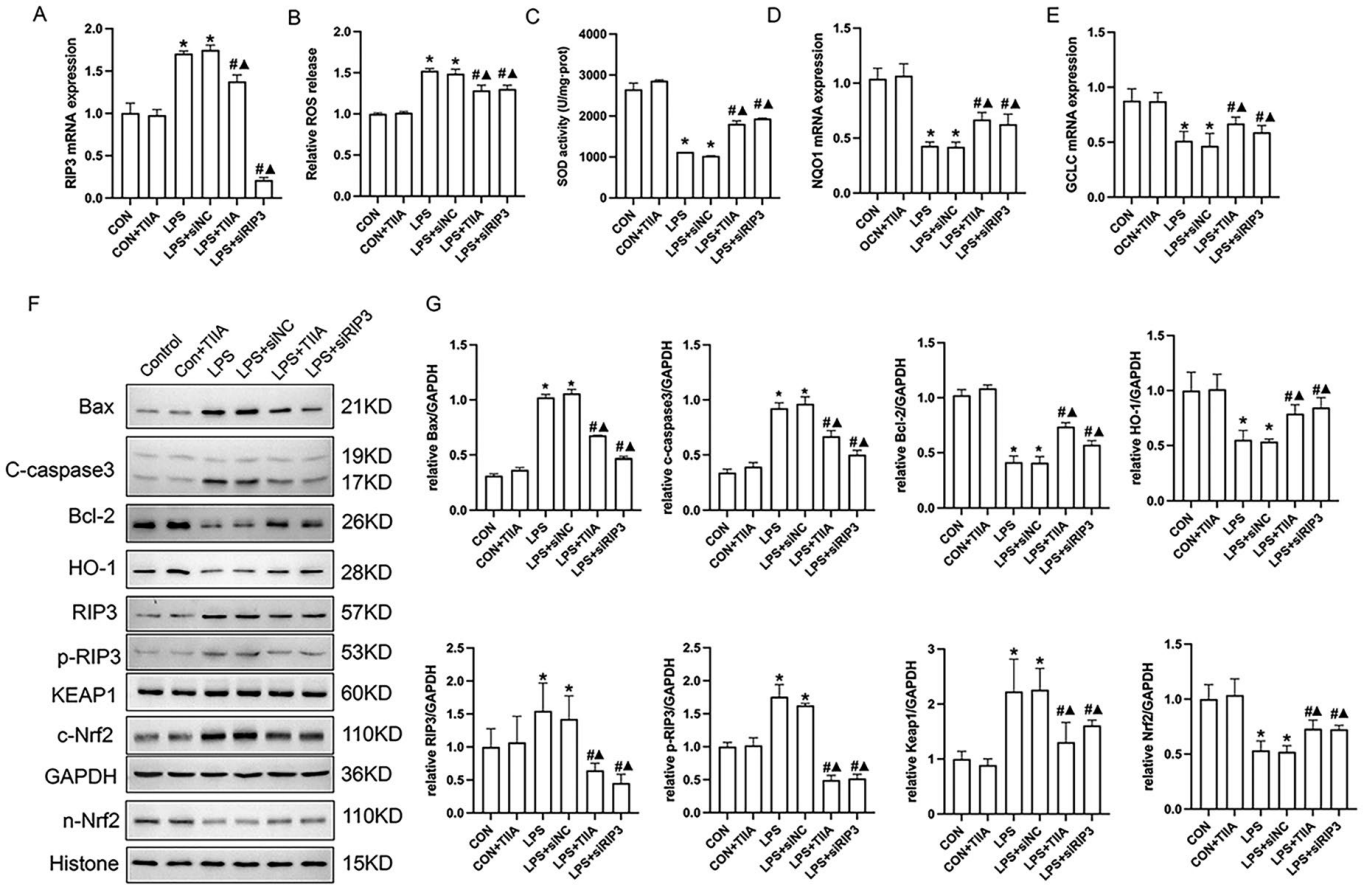
Effects of Tan IIA on oxidative stress and apoptosis *via* the RIP3/Nrf2 pathway in HK-2 cells. (A) RIP3 mRNA expression levels across groups was detected by RT-qPCR. (B and C) ROS production (B) and SOD activity (C) in HK-2 cells under different treatments. (D and E) The mRNA expression levels of NQO1 and GCLC were measured by RT-qPCR. (F and G) Western blot analysis of apoptosis-related proteins (Bax, cleaved caspase-3, and Bcl-2) and RIP3/Nrf2 pathway components (RIP3, p-RIP3, HO-1, KEAP1, and cytoplasmic Nrf2 (c-Nrf2) and nuclear Nrf2 (n-Nrf2)). *P < 0.05 vs. Control; #*P* < 0.05 vs. LPS group; Δ*P* < 0.05 vs. LPS+siNC. LPS, lipopolysaccharide; Tan IIA, Tanshinone IIA; RIP3, receptor-interacting protein kinase 3; ROS, reactive oxygen species.

To further explore the molecular mechanisms underlying the protective effects of Tan IIA observed *in vivo*, an *in vitro* model using HK-2 cells was established. HK-2 cells were treated with LPS to induce oxidative stress and apoptosis, mimicking the conditions of acute kidney injury. Tan IIA treatment significantly decreased RIP3 mRNA expression and ROS production while restoring SOD activity. Notably, RIP3 knockdown *via* siRNA (LPS+siRIP3) achieved similar effects, further confirming RIP3′s role in mediating oxidative damage (Figure 4A–C).

Additionally, LPS treatment led to a marked downregulation of antioxidant genes NQO1 and GCLC (Figure 4D,E). This was accompanied by upregulation of RIP3/p-RIP3 and KEAP1, along with accumulation of cytoplasmic Nrf2 and decreased nuclear Nrf2 and HO-1 expression, indicating impaired Nrf2 nuclear translocation (Figure 4F,G). Treatment with Tan IIA or RIP3 siRNA reversed these changes, promoting Nrf2 nuclear translocation and restoring the expression of its downstream antioxidant targets.

**Figure 5.**
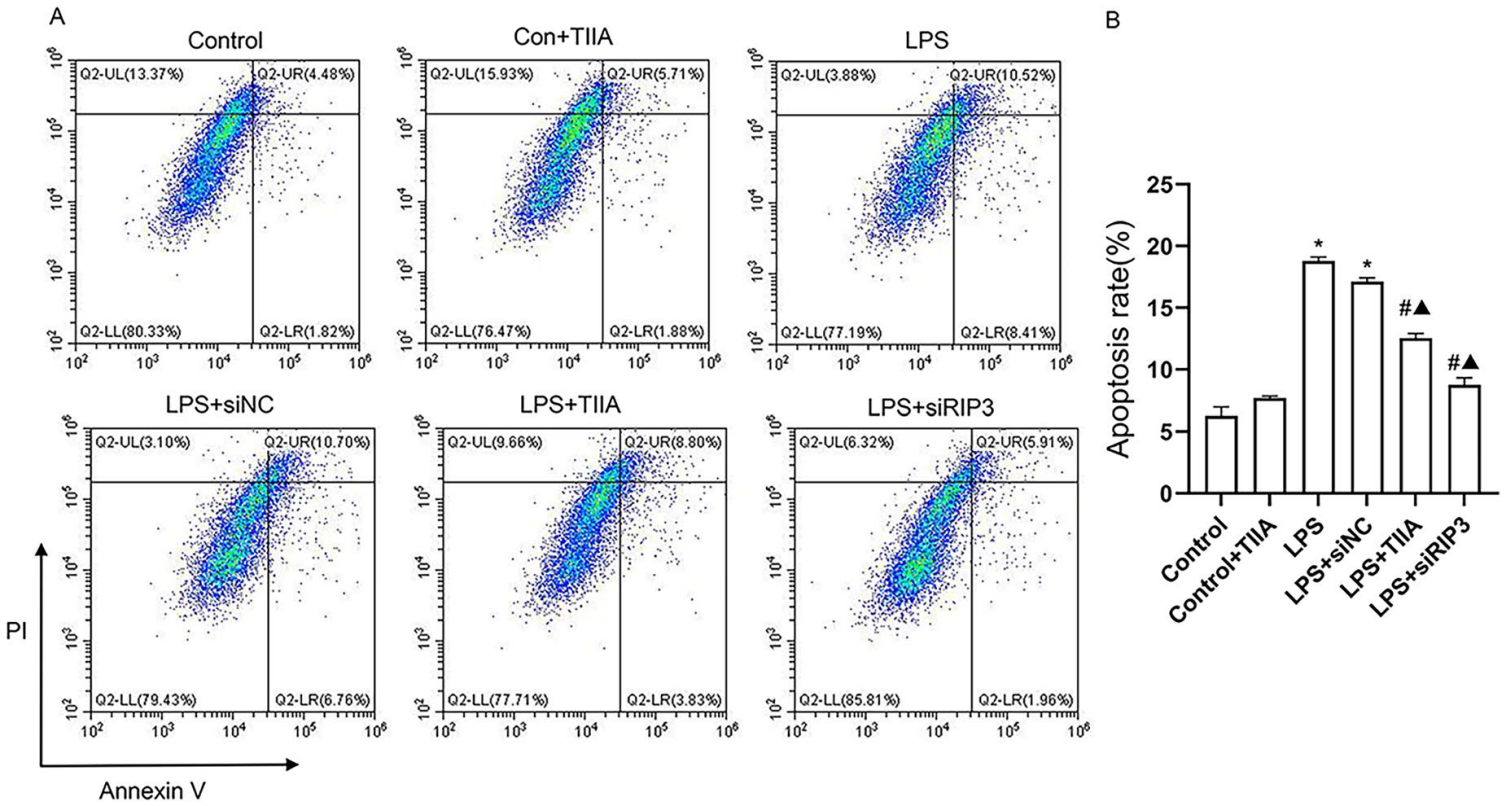
Tanshinone IIA attenuates LPS-induced apoptosis in HK-2 cells as assessed by flow cytometry. (A) Representative flow cytometry plots of Annexin V–FITC/PI double staining in HK-2 cells from each group (Control, Con + TIIA, LPS, LPS+siNC, LPS+TIIA, and LPS+siRIP3). (B) Quantitative analysis of apoptosis rate (% of total cells). *P < 0.05 vs. Control; #*P* < 0.05 vs. LPS group; ▲*P* < 0.05 vs. LPS+siNC. LPS: lipopolysaccharide; Tan IIA: Tanshinone IIA; siRIP3: small interfering RNA targeting RIP3.

Consistent with these findings, Figure 5 demonstrates that LPS significantly increased apoptosis in HK-2 cells, as shown by Annexin V/PI flow cytometry and elevated expression of pro-apoptotic proteins Bax and cleaved caspase-3, while anti-apoptotic Bcl-2 was decreased (Figures 4F,G and 5A,B). Tan IIA or RIP3 knockdown suppressed apoptosis by modulating these protein levels and reducing Annexin V-positive cells.

Collectively, these results suggest that Tan IIA protects HK-2 cells from oxidative stress and apoptosis by inhibiting RIP3 and enhancing Nrf2-mediated antioxidant responses.

### RIPK3 inhibited the nuclear translocation of Nrf2 and interacted with Nrf2 during septic AKI

**Figure 6.**
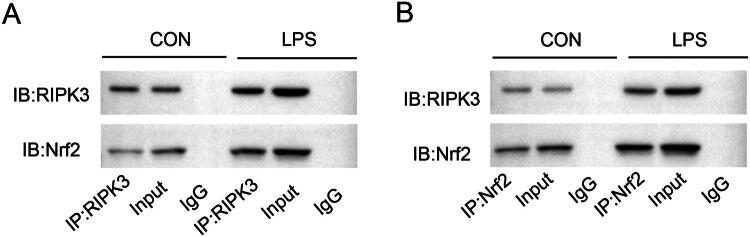
The correlation between RIP2 and Nrf2 in LPS treated HK-2 cells. Lysates from cultured HK-2 treated with LPS for 24h were subjected to immunoprecipitation using an anti-RIP3 antibody (A) or anti-Nrf2 antibody (B), and IgG antibody followed by immunoblot for Nrf2 and RIP3. Input proteins were detected with anti-RIP3 and anti-Nrf2 antibody. RIP3 interacted with Nrf2 in LPS treated HK-2 cells compared to the control group.

The further explore whether there is an interaction between RIP3 and Nrf2, we through the CoIP experimental confirmed that in the LPS induced HK-2 cells, there existed an interaction between RIP3 and Nrf2, a phenomenon not observed in the control group (Figure 6A,B).

## Discussion

This study provides new insights into the protective effects of Tan IIA against septic AKI, focusing on its ability to modulate the RIP3/Nrf2 signaling pathway. Our findings demonstrate that Tan IIA significantly ameliorates renal dysfunction and histopathological damage in a LPS-induced AKI model. This renoprotective effect is associated with the inhibition of RIP3 expression and the activation of the Nrf2 signaling pathway, leading to reduced oxidative stress and apoptosis. These findings highlight the potential clinical utility of Tan IIA as a therapeutic agent for mitigating oxidative stress and apoptosis in septic AKI.

Tan IIA administration resulted in a marked reduction in serum creatinine and blood urea nitrogen levels, indicating improved renal function. Histological analysis revealed attenuation of tubular injury, including decreased tubular necrosis and cast formation. These results are consistent with previous studies demonstrating the nephroprotective effects of Tan IIA in various models of renal injury. For instance, Tan IIA has been shown to attenuate renal injury during hypothermic preservation by reducing oxidative stress and apoptosis [10].

Tan IIA significantly mitigated oxidative stress by increasing SOD activity and reducing ROS levels, as well as 8-OHdG-positive staining in renal tissues. This finding is consistent with evidence that Nrf2 activation by Tan IIA promotes antioxidant defense and reduces oxidative damage [21,22]. Previous studies have indicated that the activation of Nrf2, a master regulator of antioxidant defense, helps mitigate renal damage in various models of kidney injury [23]. For instance, Liang et al. [11] reported that Tan IIA activates Nrf2 and increases the expression of key antioxidant enzymes such as HO-1 and NQO1, which effectively reduces oxidative damage in the kidneys. Importantly, we also observed upregulation of Nrf2 target genes (HO-1, NQO1, and GCLC), further supporting its role in antioxidant defense. These findings align with reports that Nrf2 activation plays a crucial role in mitigating oxidative stress in AKI [24,25]. Moreover, pharmacological activation of Nrf2 has been shown to protect against renal ischemia-reperfusion injury by enhancing antioxidant defenses [26].

Furthermore, the reduction in apoptosis, observed through TUNEL staining, emphasizes the role of Tan IIA in preventing cell death in the kidneys. Our results are consistent with studies showing that Tan IIA exerts anti-apoptotic effects in other models of oxidative stress. For instance, Tan IIA has been shown to reduce apoptosis and oxidative stress in cardiac, hepatic, and cerebral tissues [27,28]. The ability of Tan IIA to decrease Bax and cleaved caspase-3 levels, while increasing Bcl-2 expression, further supports its protective effects against apoptosis, reinforcing its potential as an effective therapeutic agent for renal injury. Mechanistically, Tan IIA downregulated RIP3 and its phosphorylated form (p-RIP3) while promoting Nrf2 nuclear translocation. In addition, we have confirmed an interaction between RIP3 and Nrf2. These findings corroborate studies indicating that RIP3 is a central mediator of necroptosis and oxidative damage in AKI [13,14,29]. Furthermore, Sureshbabu et al. demonstrated that RIP3 inhibition ameliorates renal injury by reducing mitochondrial dysfunction and apoptosis [15]. Our study highlights the dual role of RIP3 and Nrf2 in the apoptotic and oxidative stress pathways, adding a novel dimension to the mechanistic understanding of Tan IIA’s effects.

While our study provides valuable insights into the protective mechanisms of Tan IIA in septic AKI, several limitations should be acknowledged. First, the study was conducted in an animal model, and the translational relevance to human AKI requires further investigation. Second, we focused primarily on the RIP3/Nrf2 pathway; other pathways involved in AKI pathogenesis, such as inflammation and autophagy, were not explored. Future studies should aim to elucidate the comprehensive network of signaling pathways modulated by Tan IIA. Additionally, dose-response studies and long-term evaluations are necessary to determine the optimal therapeutic window and potential side effects of Tan IIA.

## Conclusion

In conclusion, Tanshinone IIA exhibits significant protective effects against septic acute kidney injury by inhibiting RIP3 expression and activating the Nrf2-mediated antioxidant response, thereby reducing oxidative stress and apoptosis. These findings suggest that Tan IIA holds promise as a therapeutic agent for the treatment of septic AKI. Further clinical studies are warranted to validate its efficacy and safety in human subjects.

## Data Availability

The datasets analyzed during the research are available upon reasonable request from the corresponding author.
